# Three-Month cART Initiated During Primary HIV Does Not Correct the Structural, Immune, and Microbial Abnormalities within the Gastrointestinal Tract

**DOI:** 10.20411/pai.v10i2.864

**Published:** 2025-11-13

**Authors:** Camilla Tincati, Valeria Bono, Silvia Nozza, Alessandra Bandera, Delfina Tosi, Valentina Sala, Giuseppe Ancona, Andrea Calcagno, Antonio Muscatello, Stefano Rusconi, Matteo Augello, Roberta Rovito, Umberto Gianelli, Carlo Pescia, Andrea Santoro, Monica Falleni, Andrea Gori, Giulia Marchetti

**Affiliations:** 1 Clinic of Infectious Diseases, Department of Health Sciences, ASST Santi Paolo e Carlo, University of Milan, Italy; 2 Infectious Diseases Unit, IRCCS San Raffaele Scientific Institute, Milan, Italy; 3 Foundation IRCCS Ca' Granda Ospedale Maggiore Policlinico, Infectious Diseases Unit, Milan, Italy; Department of Pathophysiology and Transplantation, University of Milan, Milan, Italy; 4 Unit of Pathology, Department of Health Sciences, ASST Santi Paolo e Carlo, University of Milan, Italy; 5 Infectious Diseases Unit, Department of Biomedical and Clinical Sciences, ASST Ovest Milanese, Ospedale di Legnano, University of Milan, Italy; 6 Department of Medical Sciences, Unit of Infectious Diseases, Amedeo di Savoia Hospital, University of Turin, Turin, Italy; 7 Department of Infectious Diseases, Luigi Sacco Hospital, ASST Fatebenefratelli Sacco, Milan, Italy; Department of Biomedical and Clinical Sciences, University of Milan, Milan, Italy

**Keywords:** Acute HIV, Gut Damage, Mucosal Immunity, Mucosal Microbiome

## Abstract

**Background:**

HIV infection leads to profound alterations of gut structure, immunity, and microbiome, resulting in immune activation and inflammation, which drive the development of non-infectious comorbidities. The introduction of combination antiretroviral therapy (cART) in the chronic stages of disease does not correct such abnormalities; however, the effect of viro-suppressive treatment in the gastrointestinal tract during primary HIV infection (PHI) is largely unknown. We studied the effects of 12-week cART on gastrointestinal (GI) structure, immunity, and mucosal microbiome in people living with HIV (PLWH) with PHI.

**Methods:**

Eleven participants with PHI enrolled in the INACTION trial underwent colonoscopy with ileum and colon biopsies, as well as peripheral blood mononuclear cell (PBMC) and plasma collection, prior to and at 12 weeks of cART. Gut biopsies were stained with CD14, CD68, CD163, and E-cadherin antibodies and Masson trichrome. Flow cytometry was performed on lamina propria and PBMCs to characterize CD4, γδ T, Treg, and Th17 cells. Gut tissue-associated microbiome analysis was conducted on colon and ileum biopsies. Ten untreated individuals with chronic HIV infection (CHI) were also studied for comparative analysis.

**Results:**

Despite treatment of PHI, gut barrier damage (E-cadherin loss, collagen deposition) progressed, with a partially preserved distribution of intestinal macrophages. Treated PHI showed stable CD4+ and γδ T-cell frequencies and decreased activation of these subsets in the colon, with no effect on intestinal Th17 and Treg cells. No major changes in peripheral inflammation and intestinal barrier integrity markers were observed. Gut tissue-associated microbiome composition evolved during cART treatment in PHI.

**Conclusion:**

Despite early initiation, 12-week cART is unable to correct the HIV-mediated gut damage. Since gut injury drives systemic inflammation, which in turn fosters the pathogenesis of non-communicable comorbidities, our findings provide pathogenetic evidence of limited efficacy of early cART in reverting the HIV-associated pro-inflammatory signature and clinical risk.

## INTRODUCTION

Combination Antiretroviral Therapy (cART) has radically changed the course of HIV disease: indeed, if initiated in the early stages of chronic infection when CD4+ T-cell counts are preserved (> 500/µL), people living with HIV (PLWH) have a comparable life-expectancy to that of their uninfected counterparts [1, 2].

Despite these advances, approximately half of newly diagnosed PLWH present in later stages of infection, ie, with CD4+ T-cell counts below 350/µL, with repercussions on immune reconstitution and clinical outcome [3].

From a pathogenic standpoint, the hampered CD4+ T-cell gain and the emergence of non-communicable diseases in the context of viro-suppressive cART are closely linked to the persistence of abnormalities within the gastrointestinal (GI) tract [4, 5]. Indeed, gut barrier damage, inflammation, and fibrosis, as well as mucosal immune dysfunction, are associated with increased GI permeability and microbial translocation [4, 5]. These represent known drivers of peripheral immune activation/inflammation [4, 5], which in turn are well-established causes of poor immunological gain [6] and non-infectious comorbidities on cART [7–9]. Modifications of the blood and gut microbiome further contribute to these outcomes in treated PLWH [10–14] by influencing systemic inflammation and cellular metabolic functions [15–17].

In line with literature reports of worse clinical outcomes in PLWH presenting in advanced stages of infection, prior studies demonstrated that the degree of gastrointestinal impairment, gut-dependent inflammation, and dysbiosis are linked to the CD4+ T-cell nadir [18–21]. More recently, however, we showed that PLWH with primary/acute HIV infection (PHI/AHI) display mucosal alterations resembling those of chronic disease, vis-à-vis lower levels of microbial translocation [22]. This raises the question as to whether the introduction of cART in PHI is able to arrest the progression of, if not to revert, the structural and functional abnormalities within the GI tract. In this respect, the resolution of gut inflammation in the lamina propria was found in one study [23], and the partial amelioration of mucosal activation as well as Th17 frequencies and function was reported in another [24]. Following these findings, enduring enterocyte turnover, monocyte activation, and fibrosis biomarkers in the peripheral blood were reported following treatment of PHI [25] and were hypothesized to be linked to the early establishment of gut dysbiosis [26]. To reconcile these diverging findings, we conducted a comprehensive study on the effects of 12-week cART on GI structure, immunity, and mucosal microbiome in PLWH with PHI.

## MATERIALS AND METHODS

### Study Population

The study population included participants enrolled in the immunological substudy of the INACTION trial, in which newly-diagnosed PHI were randomized to 3 different cART regimens [27]. The study was approved by the Milan Area 1 Ethical Committee (approval # 13547/2018, approval date 12/03/2018; EudraCT # 2017-000554-19). Diagnosis of PHI was established on the basis of a positive p24 antigenemia or detectable HIV-RNA with a negative/indeterminate Western Blot confirmation assay; staging of HIV infection was made according to Fiebig classification [28].

Upon providing written informed consent, individuals were enrolled, their demographic and clinical characteristics recorded, and peripheral blood samples collected for plasma and PBMCs separation prior to (T0) and following 12 weeks (T12) of cART. At the same time-points, individuals underwent colonoscopy at the Endoscopy Unit of San Paolo Hospital in Milan, Italy, for tissue sampling and subsequent immunohistochemical/flow cytometric experiments (see below).

Chronically HIV-infected (CHI) and cART-naïve, late-presenter individuals were enrolled as controls at the Clinic of Infectious Diseases at the same institution. In this group, laboratory analyses were only performed before cART introduction (T0), as repeated colonoscopies and plasma/PBMCs samples at T12 were not available.

### Colonoscopy

Participants belonging to the PHI group underwent repeated colonoscopies at T0 and T12 as part of the INACTION immunological substudy, while CHI individuals received a basal colonoscopy as a routine screening procedure offered at diagnosis of HIV infection.

Before the procedure, patients were asked to receive bowel preparation with Moviprep – Norgine. Colonoscopy was performed at the Endoscopy Unit of San Paolo Hospital. Standard sedation with a combination of midazolam (2mg) and pethidine (50mg) during the examination.

Two pinch biopsies were collected in the colon and 2 in the ileum: from each site, 1 was processed for flow cytometric analysis, and 1 was fresh-frozen. Additionally, biopsies from all colonic tracts and distal ileum were obtained (1 biopsy per site) and processed for routine histopathological examination, after standard fixation for 24 hours.

### Immunohistochemical Staining

Colonic biopsies were routinely formalin-fixed and paraffin-embedded, and serial 3 µm-thick sections were performed from each block; morphological signs of inflammation, integrity of intestinal barrier, and collagen deposition were evaluated in areas with oriented tubular glands in at least 5 interglandular spaces. Immunohistochemistry was performed using the Autostainer Dako Omnis. The following antibodies were used: anti-CD14, clone EPR 3653 (dilution 1:25), Gennova Scientific; anti-CD68, clone PG-M1 (ready to use), Dako Agilent; anti-CD163, clone 10D6 (dilution 1:100), Gennova Scientific; anti-E-Cadherin, clone NCH-38, (ready to use), Dako Agilent.

CD14, CD68 (PGM-1), and CD163 antibodies were used to assess the monocyte-macrophage lineage. We analyzed and quantified these populations because their role in gut inflammation in HIV, and their relationship with stromal fibrosis, has been only partially characterized—particularly with respect to the M2 (CD163+) subpopulation [29]. We used ImageJ visualization tool to accurately assess the percentage of monocytic-macrophagic cells out of 100 counted interstitial cells across 5 intervillar spaces divided into 3 virtual compartments along the glandular axis (up-per, mid, and lower third).

Integrity of the intestinal barrier was evaluated with E-cadherin antibody (CDh)1, based on a previously published semi-quantitative score [22] using a semi-quantitative score as follows: 1 = no reduction of membranous staining; 2 = reduction in the luminal surface with retained basolateral expression; 3 = lateral positivity only; 4 = basal positivity only.

Lastly, collagen stromal deposition was evaluated with Masson trichrome staining (Bio-Optica Spa) and quantified in 3 non-overlapping 40× areas on lamina propria by ImageJ. The lamina propria was outlined to define the region of interest (ROI), threshold set to omit background, and fibrosis was calculated as the percentage of unmasked pixels above threshold, relative to total pixels within the ROI. Data are presented as % of fibrotic area [22].

For immunohistochemical staining experiments, biopsies from people living without HIV undergoing routine colonic screening (n = 5) were used as historical controls.

### Isolation of Lamina Propria Mononuclear Cells

Biopsies were digested at 37°C for 1 hour in 0.25mg/mL of collagenase D (Roche) and 300 μg/mL DNase (Sigma) in complete RPMI. Cells were filtered through a 50-micron filter, washed with 5 mL 1X PBS, and pelleted. Cells were then resuspended in HBSS (Euroclone) and centrifuged at 1500 rpm for 5 minutes at room temperature. Mononuclear cells were collected at the interface and used for subsequent flow cytometry analyses (see below).

### Flow Cytometry

PBMCs were isolated from EDTA tubes collected venous blood using Ficoll-Histopaque (Biocoll, BIOSPA), cryo-preserved in fetal bovine serum (EuroClone) with 10% Dimethyl Sulfoxide (EuroClone), then stored in liquid nitrogen. For the surface phenotyping, PBMCs were thawed, and 1.5 × 10^6^ cells were plated for 3 hours in complete RPMI containing 10% human serum supplemented with 1% Penicillin–Streptomycin–Glutamine. Rested PBMCs were then stained for 30 min at 4°C in the dark. The following antibodies were used for surface immunophenotyping: CD3 (V500), CD4 (APC), TCR-γδ (FITC), CD38 (PE), Vδ1 (APC), Vδ2 (PE-Cy7), CD161 (PerCP-Cy5.5), CCR6 (PE-Cy7), CD127 (FITC), CD25 (V450) (Miltenyi, Biotech); dead cells were labeled using LIVE/DEAD Fixable cell stain APC-H7 (Molecular Probes, Life technologies). Combinations used were: CD4+ CCR6+ CD161+ (Th17), CD3+ pan-TCRγδ+ (γδ T cells), CD4+ CD25+ CD127-(Treg cells), CD3+ TCRγδ+ CD38+ (γδ T-cells activation), CD4+ CD38+ (CD4 T-cells activation).

A 2% paraformaldehyde (Sigma-Aldrich) in 1X PBS and 4% bovine serum albumin (Sigma-Aldrich) in 1X PBS were used for cell fixation and blocking, respectively. Data acquisition was performed using FACSVerse™cytometer (BD Biosciences) and thereafter analyzed using FlowJo software 10.7.2 (BD Biosciences). For lamina propria mononuclear cells immunophenotyping, freshly isolated cells, as previously described, were submitted directly to cell staining, fixation, and data acquisition using the same panel of PBMCs FACS analysis. An example of the gating strategy used is presented in Supplementary Figure 1.

### Plasma Biomarkers Quantification

Plasma cytokine Interleukin-6 (IL-6) was quantified using Human Quantikine HS ELISA kit (R&D Systems, Inc.), E-cadherin, and soluble (s) CD14 were quantified with Human Quantikine ELISA kits (R&D Systems, Inc.) according to the manufacturer's instructions. Freshly thawed plasma samples were diluted 1:20, 1:200 with the calibrator diluent for E-cadherin and sCD14 ELISA kits, respectively. EndoCab IgG Human, Human lipolysaccharide binding protein (LBP), Human 1,3-beta-D-glucan, and I-FABP markers were measured by ELISA (EndoCab IgG cod HYC-HK504-IGG, Hycult Biotech; LBP cod E0360HU-96; 1,3-beta-D-glucan cod E4111hu-96, BT LAB; Human I-FABP Immunoassay cod DFBP20, R&D Systems), following the manufacturer's instructions. The optical density (OD) of each well was measured using the microplate reader set to 450 nm (EnSight™ multimode plate reader, PerkinElmer Inc.).

### Gastrointestinal Mucosal Microbiome Analysis and Bioinformatics

Colon and ileum biopsies were stored at −80°C. DNA was extracted from the samples using tissue-specific optimized techniques at Vaiomer SAS. The integrity and concentration of DNA were evaluated using UV spectroscopy (Nanodrop^^®^^, Thermo Scientific).

### Library Preparation

PCR amplification was performed using 16S universal primers targeting the V3-V4 region of the bacterial 16S ribosomal gene (Vaiomer universal 16S primers). The joined pair length was set to encompass a 467 base pair amplicon using the 2 × 300 paired-end MiSeq kit V3. For each sample, a sequencing library was generated by addition of sequencing adapters.

### Sequencing

The detection of the sequencing fragments was performed using the MiSeq Illumina^®^ technology. The sequencing output target was 75,000 raw reads (2 × 37,500 paired-end reads) per sample, which has been experimentally determined to be the number of reads to have exhaustive coverage of the community profiles present in high diversity samples. A variation in the total number of reads (up to 2-fold change) can occur between samples and is considered acceptable for analysis.

### Bioinformatic Analysis

The targeted metagenomic sequences from microbiota were analyzed using the bioinformatics pipeline established by Vaiomer [30, 31]. Briefly, after demultiplexing of the bar-coded Illumina reads, single read sequences were cleaned and paired, for each sample independently, into longer fragments. After quality-filtering (abundance, fragment length and sample quality), the data were clustered into operational taxonomic units (OTU) using the Swarm algorithm, a method which allows the clustering threshold to adapt to the input data. Then, a taxonomic assignment was performed in order to determine community profiles.

### Statistical Analysis

Descriptive and statistical analyses were performed with the use of GraphPad Prism 9.0 (GraphPad Inc.). Discrete variables are presented as absolute/percentage values and analyzed by Fisher's exact test. Continuous variables are presented as median values and interquartile range and analyzed with the Wilcoxon and Mann-Whitney tests. A *P* value < 0.05 was considered statistically significant.

## RESULTS

### Study Population

A total of 21 participants were enrolled in the study (PHI, n = 11; CHI, n = 10). The 2 groups had comparable demographic characteristics (Table 1); however, at T0, PHI presented significantly higher CD4+ (538/µL [IQR, 49–609] vs 147/µL [IQR, 12–279]; *P*=0.001) and CD8+ T-cell counts (1352/µL [IQR, 826–1752] vs 688/µL [IQR, 248–925]; *P*=0.02) as well as a trend to higher HIV RNA (5.58 log10 cp/mL [IQR,5.16–5.92] vs 5.13 log10 cp/mL [IQR, 4.8-5.4]; *P*=0.05) than CHI (Table 1).

**Table 1.** Demographic and Clinical Features of the Study Population

	PHI (N = 11)	CHI (N = 10)	*P* value
**Age at enrollment [years], median (IQR)**	41 (29-45)	42 (34-51)	0.34
**Male sex, n (%)**	11 (100)	8 (80)	0.21
**Ethnicity, n (%)**			
Caucasian	10 (91)	7 (70)	
Other	1 (9)	3 (30)	0.31
**Epidemiology, n (%)**			0.59
MSM	10 (91)	8 (80)	
Heterosexual/other	1 (9)	2 (20)	
**HBV/HCV coinfection, n (%)**	3 (27)	3 (30)	1
**CD4+ T cells at enrollment**			
cells/µL, median (IQR)	538 (493-609)	147 (12-279)	0.001
%, median (IQR)	25 (14-28)	17 (10-18)	0.13
**CD8+ T cells at enrollment**			
cells/µL, median (IQR)	1332 (826-1752)	688 (248-925)	0.02
%, median (IQR)	56 (49-72)	58 (49-66)	0.53
**CD4/CD8 ratio at enrollment, median (IQR)**	0.50 (0.19-0.57)	0.26 (0.15-0.32)	0.17
**HIV-RNA at colonoscopy [median log10 cp/mL, IQR]**	5.58 (5.16-5.92)	5.13 (4.80-5.42)	0.05
**Fiebig stage, n (%)**			
I	0 (0)		
II-III	4 (36)	NA	NA
IV-V	7 (63)		
**cART treatment (INACTION trial), n (%)**			
TAF/FTC/DRVc	3 (27)	NA	NA
TAF/FTC+ DTG	6 (54)		
TAF/FTC/DRVc +DTG	2 (18)		
**CDC stage, n (%)**			
C1-C3 (AIDS-defining conditions)	1 (9)	4 (40)	0.15
Other	10 (91)	6 (60)	

MSM: Men having Sex with Men; HBV/HCV: Hepatitis B Virus/Hepatitis C Virus; NA: Not applicable

Among PHI individuals, 4/11 were diagnosed in Fiebig stages II-III and 7/11 in stages IV-V, with an estimated time of infection of 30 days and 40 days, respectively [28]. cART was introduced at a median time of 12 days (IQR 9–26) from HIV diagnosis in PHI. Upon cART introduction in PHI, we found a significant increase in total (756/µL [IQR, 590–940]; *P*=0.004) and percentage (37.7% [IQR, 33–43.7]; *P*=0.001) CD4+ T cells as well as a rise of CD4+/CD8+ T-cell ratio (1.23 [IQR, 0.76–1.44]; *P*=0.001); parallelly, we observed a decrease in total (816/µL [IQR, 554–900]; *P*=0.001) and percentage (35% [IQR, 28.4–40.7]; *P*=0.001) CD8+ T cells and HIV RNA levels (1.6 log10 cp/mL [IQR, 1.55–1.91]; *P*=0.001).

### Progressive impairment of the gut barrier is observed despite early cART in PHI

**Figure 1.**
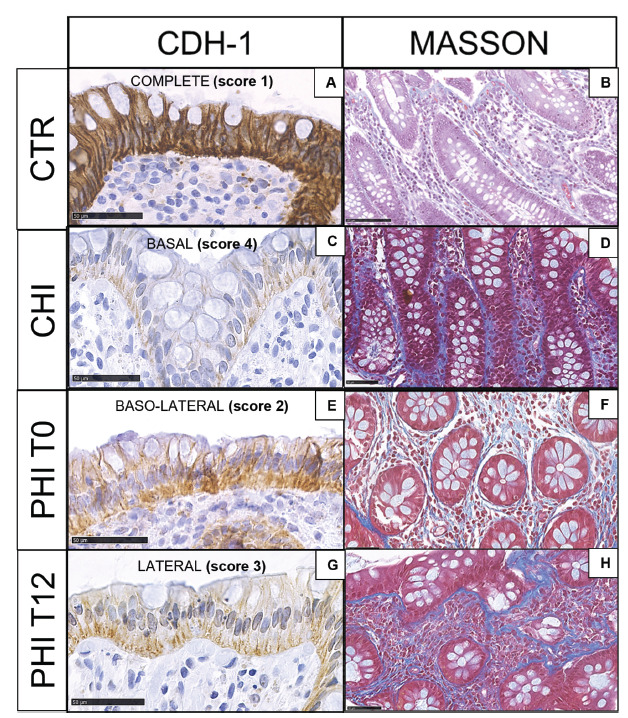
**E-cadherin and Masson trichrome staining in colonic biopsies.** A,B) HIV-uninfected controls (CTR) show epithelial colonic cells, which are highly immunoreactive to E-cadherin (complete expression, score 1) and faint blue collagen fibers in the stroma; C,D) Individuals with chronic HIV infection (CHI) feature the loss of E-cadherin immunostaining (basal expression, score 4) and heavy fibrosis; E,F) Individuals with untreated (T0), primary HIV infection (PHI) show loss of E-cadherin immunoreactive cells (baso-lateral expression, score 2) and collagen deposition; G,H) Individuals with PHI following 12 weeks of cART (T12) display progressive E-cadherin loss (lateral expression, score 3) and collagen deposition.

As compared to HIV-uninfected controls (Figure 1A-B), CHI displayed marked reduction of E-cadherin expression with residual immunoreactivity at the bottom of the glands (Figure 1C) as well as a massive deposition of collagen fibers (Figure 1D). In contrast, in PHI at T0, a decrease in E-cadherin immunostaining was detected from the luminal surface to the lowest part of the gland (Figure 1E), and mildly/intense collagen fibers were observed (Figure 1F). At T12, E-cadherin expression was progressively lost, with the highest depletion in the upper third of the gland (Figure 1G) and detection of dense fibrosis (Figure 1H). Detailed results for E-cadherin expression and Masson staining are presented in Supplementary Table 1A-B. We did not observe significant variations in neutrophil infiltration across different samples.

### Intestinal macrophages are partially maintained during early cART in PHI

**Figure 2.**
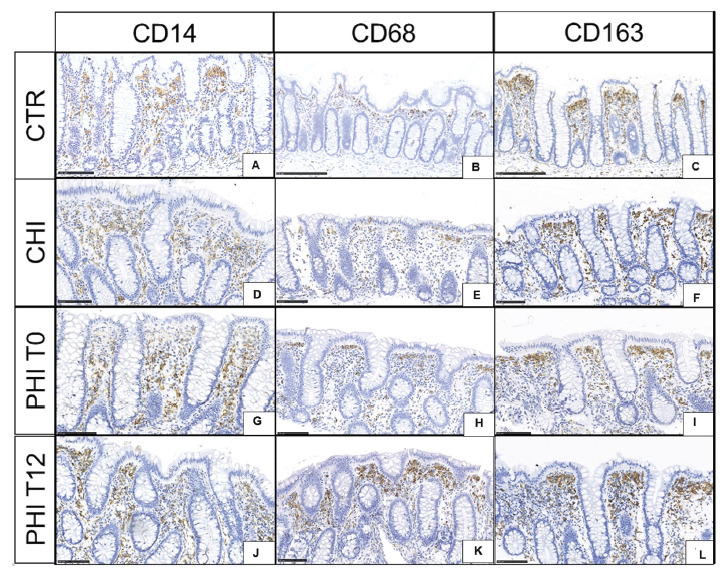
**CD14, CD68, and CD163 immunoreactive cells in colonic biopsies.** A,B,C) HIV-uninfected controls (CTR) display homogeneous distribution of CD14+ immunoreactive cells with a subepithelial reinforcement of CD68+ and CD163+ cells. D,E,F) Individuals with chronic HIV infection (CHI) feature a similar number and distribution of CD14+ cells to that observed in uninfected controls, yet a mild increase of CD68+ and CD163+ cells in the subluminal stromal space. G,H,I) Individuals with untreated (T0), primary HIV infection (PHI) show recruitment of CD14+ from the mid-third of the interglandular space as well as scant increases of CD68+ and CD163+ cells. J,K,L) Individuals with PHI following 12 weeks of cART (T12) feature homogeneous distribution of stromal CD14 immunoreactive and stable CD68+ and CD163+ cells.

In HIV-uninfected controls, CD14 immunoreactive cells showed a homogeneous distribution along the interglandular space (Figure 2A), while subepithelial luminal reinforcement (“T-like” disposition) was detected for CD68+ (Figure 2B) and CD163+ (Figure 2C) cells.

CHI showed comparable distribution and number of CD14 immunoreactive cells to uninfected controls with a milder subepithelial luminal concentration (Figure 2D). However, a mild increase of CD68+ (Figure 2E) and CD163+ macrophages (Figure 2F) was noted in the upper third of the stromal space between 2 glands.

In PHI at T0, CD14+ cells increased at the mid-third of the glandular axis (Figure 2G); CD68+ and CD163+ immunoreactive cells also increased, yet maintained a similar distribution to that of uninfected controls (Figure 2H, I). At T12, CD14+ cells showed a homogeneous distribution in the interglandular space (Figure 2J), while CD68+ and CD163+ cells were unaffected (Figure 2K, L).

Detailed results for macrophage populations across samples are provided in Supplementary Table 1C, along with a visual representation of the range of monocytic/macrophagic populations (Supplementary Figure 2).

### Treated PHI show stable CD4 T-cell counts and decreased activation in the colon

**Figure 3.**
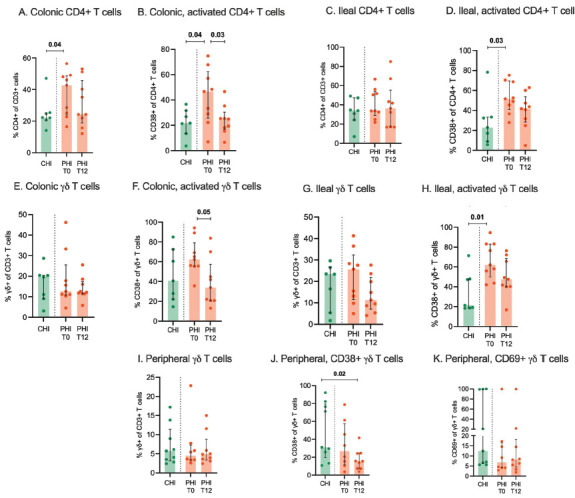
**CD4+ and Υδ T cells in colon, ileum and peripheral blood.** Total and activated CD4+ and Υδ T cells were measured by flow cytometry in colonic (A-D) and ileal tissue biopsies (E-H) as well as peripheral blood (I-K) from individuals with chronic HIV infection (CHI, green) and primary HIV infection (PHI, orange) prior to (T0) and following 12-week cART (T12).

In the colon, at T0, PHI showed significantly higher total (42.7% [IQR, 24–48.6] vs 22.3% [IQR, 20.7–25.4]; *P*=0.04; Figure 3A) and activated, CD38-expressing CD4+ T cells than CHI (46.7% [IQR, 26.7–62.4] vs 21.7% [IQR, 13.5–31.7]; *P*=0.04; Figure 3B). At T12, despite no changes in total CD4+ (24.3% [IQR, 18.9–45.8]; *P*=0.07; Figure 3A), activated CD4+ T cells decreased significantly (25% [IQR, 16.8–20.4]; *P*=0.03), reaching levels comparable to those seen in CHI (Figure 3B).

In the ileum, at T0, CD4+ T-cell counts were similar in PHI and CHI (Figure 3C), yet activated CD4+ T cells were higher in PHI (51.5% [IQR, 41–69.5] vs 22.9% [IQR, 8.9–33.3]; *P*=0.03; Figure 3D). At T12, total and CD38-expressing CD4+ T-cell frequencies remained stable despite cART (Figure 3D).

### Treated PHI display stable γδ T-cell counts and a trend to reduced activation in the gut and blood

In the colon, at T0, PHI displayed total and activated γδ T-cell counts comparable to CHI (Figure 3E); at T12, the former remained stable while the latter showed a trend to significant decrease (T0: 62.2% [IQR, 55.5–79.1]; T12: 33.9% [IQR, 20.8–57.4]; *P*=0.05); Figure 3F).

In the ileum, at T0, total γδ T-cell counts were similar in the two groups (Figure 3H) while activated cells were significantly higher in PHI (62% [IQR, 50–83.1] vs 21.4% [IQR, 18.5–48]; *P*=0.01; Figure 3H) and underwent a non-statistically significant decrease following treatment (47.5% [IQR, 40.1–69]; *P*=0.09; Figure 3H).

In the peripheral blood, no statistically significant difference was observed in the frequency of γδ T cells (Figure 3I) at both time points. A significant decrease of activated CD38-expressing, yet not CD69-expressing, γδ T cells was shown in PHI at T12, reaching levels significantly lower compared to CHI subjects at T0 (14% [IQR, 7.2–15.8] vs 31.1% [IQR, 19.5–79.4]; *P*=0.01) (Figure 3J, K).

No significant differences were found in the frequency of Vδ1 and Vδ2 cells in PHI and CHI individuals in the gut or peripheral blood (not shown).

### cART has no effect on intestinal Th17 and Treg cells in PHI, yet it increases peripheral Treg levels

**Figure 4.**
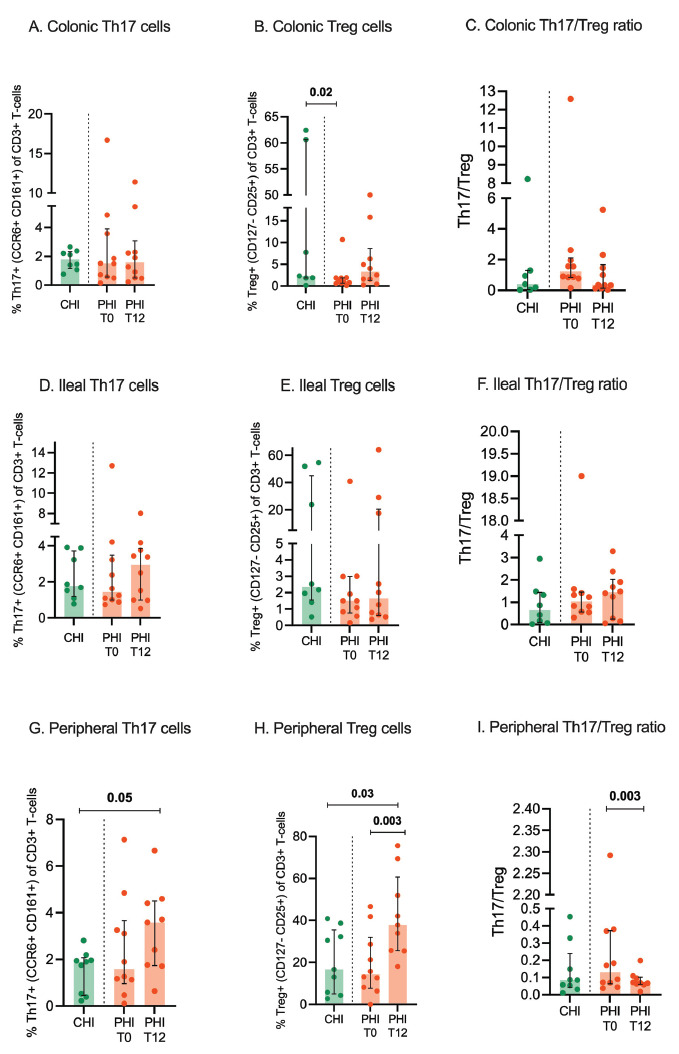
**Th17, Treg cells, and Th17/Treg ratio in colon, ileum, and peripheral blood.** Th17 and Treg cells were measured by flow cytometry in colonic (A-C) and ileal tissue biopsies (D-F) as well as peripheral blood (G-I) from individuals with chronic HIV infection (CHI, green) and primary HIV infection (PHI, orange) prior to (T0) and following 12-week cART (T12). Percentages of Th17 and Treg cells are expressed within the CD3+ T cells.

In the colon and ileum, PHI and CHI displayed comparable Th17 and Treg cells at both time-points (Figure 4A-B; D-E); higher colonic Treg cells in CHI vs PHI at T0 (2.3% [IQR, 1.8–60.6] vs 1.06% [IQR, 0.6–1.9]; *P*=0.02) were mainly driven by outliers (Figure 4B). Accordingly, the Th17/Treg ratio was comparable in CHI and PHI at mucosal sites, regardless cART introduction (Figure 4C-F).

In the peripheral blood, at T0, we also found similar Th17 and Treg cells in the two groups; at T12, a significant increase of Treg was registered in PHI (15.7% [IQR, 7.2–35.2] vs 37.8% [25.5-60.6]; *P*=0.003; Figure 4H), resulting in higher levels than CHI (16.6% [IQR, 5–35.4]; *P*=0.03; Figure 4H) as well as a contraction of the Th17/Treg ratio 0.1% [IQR, 0.07–0.3] vs 0.06% [IQR, 0.06–0.1]; *P*=0.003) (Figure 4I).

### No changes in peripheral inflammation and intestinal barrier integrity markers were observed during early cART in PHI

**Figure 5.**
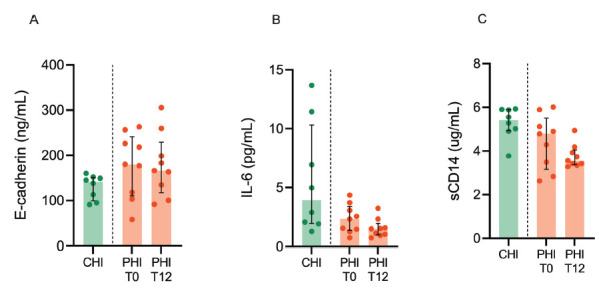
**Peripheral inflammation and intestinal barrier integrity markers.** Markers of intestinal barrier integrity (E-cadherin; A) inflammation (IL-6, interleukin-6; B), and immune activation (sCD14, soluble CD14; C) were measured in plasma from individuals with chronic HIV infection (CHI, green) and primary HIV infection (PHI, orange) prior to (T0) and following 12-week cART (T12).

We observed similar levels of plasma E-cadherin, IL-6, and sCD14 in CHI and PHI at T0 (Figure 5A-C). In addition, cART exerted no major effect on such markers, except for a trend to lower IL-6 levels (2.3% [IQR, 1.3–3.3] vs 1.5% [IQR, 0.9–1.9]; *P*=0.07) (Figure 5B). Similarly, no statistical differences were noted following cART introduction in PHI in terms of additional markers of gut barrier damage and microbial translocation (ie, I-FABP, EndoCab, LBP, and 1,3-beta-D-glucan (Supplementary Figure 3).

### Gastrointestinal Tissue Microbiome Analysis

To characterize the local microbiome in association with the mucosal tissue microenvironment, ileum and gut biopsies were analyzed.

**Figure 6.**
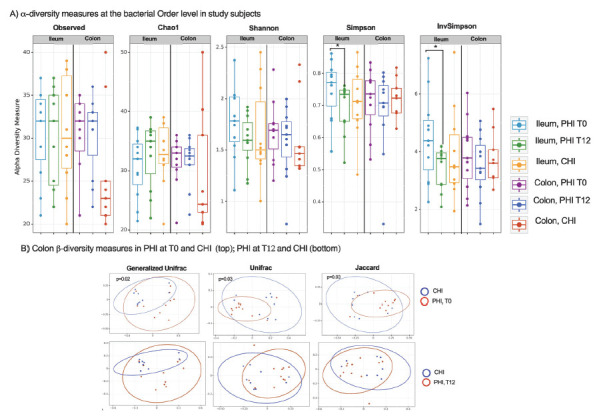
**Alpha and Beta diversity of microbial communities across samples.** A) Alpha-diversity indices at the bacterial Order level in ileum (left panel for each measure) and colon (right panel for each measure) in study participants. B) Beta-diversity indices (Principal Coordinates Analysis, PCoA) in colon biopsies, comparing (top) CHI and PHI at T0; (bottom) CHI and PHI at T12 by Permanova (pseudo-F values).

For all taxonomic levels, α-diversity measures did not vary significantly among study groups at both mucosal sites (ileum and colon) (Figure 6A). The only exception was a significant decrease of α-diversity bacterial Order in the ileum (Simpson and InvSimpson, *P*=0.04) following treatment in PHI (Figure 6A).

In addition, β-diversity measures showed significantly different colonic bacterial community composition between PHI at T0 and CHI (generalized Unifrac: *P*=0.02; Unifrac: *P*=0.03; Jaccard: *P*=0.03) (Figure 6B); these differences were lost following cART treatment with PHI displaying, at T12, similar β-diversity indices to those observed in CHI (Figure 6B).

**Figure 7.**
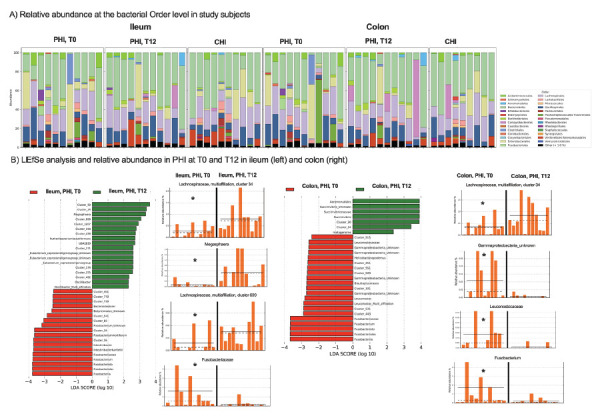
**Mucosal microbiome changes in PHI following cART in PHI.** A) Relative abundance at the bacterial Order level in ileum (left) and colon (right) in study participants. B) Linear discriminant analysis Effect Size (LEfSe) analysis shows the taxonomic differences between PHI at T0 and T12. Representative plots of microbiome changes at the 2 time-points are also shown.

Relative abundance showed that bacterial composition tended to vary by study group (ie, CHI vs PHI), rather than sample site (Figure 7A). In the ileum, cART treatment in PHI led to significant increases of *Ruminococcaceae*-UBA1819 (*P*=0.01), *Bluatia* multi-affiliation cluster 154 (*P*=0.04), *Eubacterium* (*P*=0.03), *Oscillobacter* cluster 452 (*P*=0.03), *Ruminococcus gnavus* group (*P*=0.04), *Lachnospiraceae* multi-affiliation cluster 34 (*P*=0.03) and 699 (*P*=0.04), *Megasphaera* (*P*=0.03), and *Ruthenibacterium lactatiformans* (*P*=0.01) and decreases in *Fuscsobacteriia* (*P*=0.02), *Fusobacterium* (*P*=0.04), *Fusobacteriales* (*P*=0.02), *Fusobacteriaceae* (*P*=0.02), and *Bacteroides dorei* (*P*=0.03) (Figure 7B); in the colon, cART resulted in significant increases of *Lachnospiraceae* multi-affiliation cluster 34 (*P*=0.04) and *Aeromondales* (*P*=0.04) and decreases of *Gammaproteobacteria* (*P*=0.03), *Helicobacter apodemus* (*P*=0.02), *Leuconostocaceae* (*P*=0.03), *Fusobacteriia* (*P*=0.02), *Fusobacterium* (*P*=0.02), and *Fusobacteriaceae* (*P*=0.02) (Figure 7B).

Compared to PHI at T12, CHI maintained higher proportions of *Oscillosporaceae* cluster 319 (*P*=0.04), *Subdoligranulum* (*P*=0.04), and *Ruminococcus* in the ileum (*P*=0.04); Ruminococcus torques group (*P*=0.006), *Oscillospiraceae* (*P*=0.04), and *Helicobacter apodemus* (*P*=0.04) in the colon (Supplementary Figure 4).

## DISCUSSION

The profound alterations that occur within the gastrointestinal tract are major drivers of T-cell activation and microbial translocation in HIV infection [32]. While cART introduction in chronic HIV infection is known to have a scant effect on the gut mucosa [18–20], its impact when initiated in acute HIV infection is still unclear.

Mucosal barrier damage and fibrosis are among the root causes of HIV pathogenesis [33]. In line with prior data [22, 23], we show the early onset of junctional complex impairment and collagen deposition in the gut. These changes progressed at 12 weeks of follow-up, resembling those observed in advanced, untreated individuals, thus pointing to the limited effect of short-term antiretroviral treatment in preventing tissue injury in acutely-infected PLWH. Given the non-significant and subtle changes observed in monocytic and macrophagic populations, we cannot conclude a direct implication in gut barrier disruption or stromal remodeling, although we speculate that the mild increase in CD163+ cells in T12 samples could be linked to increased stromal fibrosis [29]. Our findings of stable plasma E-cadherin levels in PHI before and after treatment also suggest ongoing protein release from epithelial junctions in the gut. Furthermore, the accumulation of macrophages expressing CD14, the LPS co-receptor, within the intestinal crypts following acute infection indicates the potential for microbial translocation from the gut to the systemic circulation very early in the course of disease. Indeed, sCD14, a surrogate marker of microbial translocation, was detected in the peripheral blood of people with untreated PHI at levels comparable to those with CHI. Following cART treatment in acute infection, CD14+ macrophages increased in the subepithelial layer, yet sCD14 moderately decreased in the blood; together with the finding of a trending decrease in IL-6, these results show that early therapy in PHI does not extinguish gut-dependent systemic inflammation in the short term.

Mucosal T-cell populations play a major role in maintaining gut barrier function and are deeply affected by HIV infection [34]. CD4+ T cells represent the major target of HIV replication and are depleted in the gut and blood in all stages of disease [35]. In our study, PHI showed higher CD4+ T-cell frequencies than CHI in the colon, yet not in the ileum, suggesting a differential effect or kinetics of HIV-induced T-cell loss at the 2 sites. However, by showing stable CD4+ T-cell frequencies in the gut over time despite timely cART start in PHI, we confirm that cART had little/no effect on mucosal immune reconstitution [24, 36, 37].

In accordance with the role played by viral replication in the direct stimulation of T-cell activation [38], PHI displayed high frequencies of activated, intestinal CD4+CD38+ cells prior to treatment. Again, cART had a limited impact on mucosal immune activation, as it accounted for a significant decrease of activated CD4+ T cells only in the colon and not the ileum. Furthermore, the frequencies of activated CD4+CD38+ cells in treated, acute infection at both GI sites were similar to those measured in advanced chronic infection, confirming the inability of treatment to fully turn off mucosal immune activation [24], which may fuel gut damage [39].

Tissue resident γδ T cells are key players in epithelial maintenance and repair [40]. In this respect, recent work in the animal model demonstrated a pathogenic link between intestinal Vδ2 T-cell dysfunction, gut barrier disruption, and a plasma inflammatory signature during early cART-treated, chronic SIV infection [41]. In the present work, we found that the frequencies of total and activated γδ T cells in the blood and gut of acutely-treated PHI were comparable to those of chronically, untreated CHI; these results suggest that cART introduction in acute or early chronic infection [41], has a minimal effect on γδ T cells, which may contribute to the establishment of a leaky gut and systemic inflammation.

Th17 and Treg cells have a significant role at mucosal surfaces by containing infection with pathogenic microorganisms [42] and exerting suppressive control over other cells [43], respectively. While cART introduction in acute SIV infection has a differential effect on Th17 and Treg imbalances in lymphoid tissues and the peripheral blood [44], in early Fiebig stages of HIV infection, it can restore mucosal Th17 frequencies and polyfunctionality [24]. Following prior data showing that intestinal Th17 numbers and function decrease by Fiebig stage [24], we show similar Th17 and Treg frequencies in the gut and blood in untreated PHI (the majority of whom were diagnosed in Fiebig stages IV-V) and CHI individuals. We also confirm that treatment of acute infection accounted for a rise in peripheral, yet not mucosal, Th17 and Treg cells, further highlighting the limited effect of cART on gastrointestinal immunity.

Lastly, cART introduction in PHI was linked to modifications of the gut microbiome, featuring comparable α- and β-diversity measures at T12 to those observed in CHI as well as changes in taxonomic composition in gut tissue. Longer follow-up is needed to understand whether these changes are linked to the progression of HIV-induced dysbiosis, possibly pointing to a limited effect of cART in restoring the microbiome tissue imbalance. Given that HIV-related dysbiosis contributes to the decreased production of short-chain fatty acids and peripheral inflammation [45], which may lead to the development of comorbidities [10] and increased mortality [45], our research may indicate the early onset of disease progression risk.

The present study has several limitations, including the relatively small sample size, short follow-up period, enrollment of PLWH with advanced Fiebig stages, flow cytometry data restricted to gut and peripheral CD4+ T-cells (due to limited sample size and PBCMs numbers), the lack of controls for peripheral/mucosal immunity and microbiome analyses, as well as treated, chronically infected PLWH. Further, the potential for biopsy sampling differences may also cause some uncertainty in the histology results.

Finally, later comparisons of GI structure and immune activation are needed to evaluate whether cART can slow disease progression in PHI and whether it differentially impacts the microbiome and mucosal immunity depending on the antiretroviral regimen. Despite these drawbacks, our findings confirm that acute HIV infection impairs gut mucosal structure, immunity, and microbiome and that early cART does not correct such alterations in the short term. Given the established role of gut integrity loss in driving systemic inflammation [46] as well as the failure of cART to prevent GI disruption and immune activation—both of which contribute to the ongoing increase in immune activation observed in treated CHI [18]—our results shed light on the mechanisms underlying the persistence of inflammatory markers in PLWH even when treated during acute infection [25, 47] and appeal for novel therapeutic strategies to preserve the gastrointestinal tract in the earliest stages of HIV.

## SUPPLEMENTARY DATA

Supplementary materials are available at the *Pathogens and Immunity* website. Supplementary data may be provided by the authors to benefit the reader. Supplementary data are not copyedited and are the sole responsibility of the authors. Questions or comments related to supplementary materials should be addressed to the corresponding author.
